# Improvements in Working Rubber

**Published:** 1874-10

**Authors:** Luis Dunbar


### IMPROVEMENTS IN WORKING RUBBER.

BY LUIS DUNBAR, D. D. S.

Why this endless talk about the Rubber Company's monopoly and our great "burden."

What has been written upon the subject of vulcanite litigation, and the elaborate arguments made to show the falsity of the company's claims upon the profession would fill a volume of proportions simply enormous, and yet no good has resulted; the company gets three recent decisions in their favor, and immediately following them come their agents for fresh plunder.

It has long since been conceded that the Cummins' claim is not for the application of rubber to dental purposes, but for the method of working it as specified in his second re-issue, No. 1904.

This has been talked of and explained at dental meetings over and over again, and improvements suggested. But to no purpose, for the profession will persist in sticking to the ruts of old time practice as far as rubber is concerned, and pass a verdict adverse to all the improvements without ever trying them. Dr. W. H. Eames, of St. Louis, suggested an improvement two years ago in his published method for working vulcanizable Gutta-percha, which is applicable alike to all vulcanizable compounds. But the profession, slow to see a good thing, took it only as connected with Gutta-percha. Many tried it with success, as far as the manner of working was concerned, producing beautiful plates; but from some defect in the compound they softened in the mouth, hence dissatisfaction and abandonment of the method and material, and return to rubber on the Cummins' method, taxation, litigation, etc.

During the past three months I have experimented with rubber upon Dr. Eames' method for Gutta-percha, and in every case have been rewarded with success. The result being a plate of uniform thickness and a perfect fit, requiring less time to finish and the trouble of separating a flask, removing a wax plate and packing entirely avoided.

For the benefit of those who are compelled to use rubber and to whom the Rubber Company's tax is a burden, I will give the method as I interpret it:

Take the model as ordinarily prepared, being careful to do all trimming before mounting the teeth, then proceed as in making a Gutta-percha try plate, using rubber instead of the former. Cut it to the size desired, warm and press down evenly over the model. To facilitate its sticking dot the model with adhesive wax, only a small quantity being used. The air chamber can be put on in the usual way.

The teeth are then ground in carefully, heated and pressed into position, being held so while they are backed up with strips of heated rubber pressad down around the pins; smooth up level with a hot knife, touching the palatal portion as little as possible; next cut a strip and press down on the outside of the gums with a knife, out of which trim and shape the rim. Recollect it is much easier to cut from soft rubber with a hot knife than to burr or scrape after being vulcanized. When everything is trimmed up smooth, run a little wax around the teeth where they are joined by the rubber, and also where the rubber joins the model to prevent the plaster from finding its way under and spoiling the work. Next place in the flask, pouring plaster over the whole, being careful to have no air holes, as the expansion of the rubber will be wasted in filling these, and failure would be the result. In difficult cases grind to a wax or Gutta-percha try plate, try in the mouth and re-arrange on the rubber, but in all cases where the impression is good and bite carefully taken, trying in will hardly be necessary, unless there is a special fulness to be given or avoided. In partial pieces trying in is not at all necessary, simply get a good impression of the natural articulation, and success is sure, for the teeth once in position can not be displaced in the process.

After allowing time for the plaster to harden, vulcanize in the usual way, and upon removing the piece the wax will be found to have been absorbed by the plaster, and the space filled up with rubber. The result being a plate that can not fail to fit if the impression was a good one. No cracked blocks, no rubber in joints, no displacement of teeth, a plate requiring no burrs and very little scraping to finish, and entire freedom from the imputation of having infringed upon any one's patent right.

Patience must characterize all experiments in this method, which is infinitely easier than the old when once learned, but will permit of no slighting. The bungler had better stick to his "Bacon" and the old way.

